# Loneliness correlates and associations with health variables in the general population in Indonesia

**DOI:** 10.1186/s13033-019-0281-z

**Published:** 2019-04-10

**Authors:** Karl Peltzer, Supa Pengpid

**Affiliations:** 1 North West University, Potchefstroom, South Africa; 2 ASEAN Institute for Health Development, Mahidol University, 25/25 Phutthamonthon Road 4, Salaya, Phutthamonton, Nakhon Pathom, 73170 Thailand

**Keywords:** Loneliness, Health variables, Adolescents, Adults, Indonesia

## Abstract

**Background:**

Loneliness has been commonly reported in high-income countries, while less is known about loneliness in Association of the Southeast Asian Nations (ASEAN) member states, in particular in Indonesia.

**Objective:**

The aim of the study was to estimate the prevalence of loneliness, its correlates and associations with health variables in a national survey in the general population in Indonesia.

**Methods:**

In the Indonesia Family Life Survey (IFLS-5) in 2014–2015, 31,447 participants 15 years and older (median age 35.0 years, interquartile range = 22.0) were interviewed and examined in a national population-based cross-sectional study. The self-reported prevalence of loneliness, blood pressure, body height and weight, physical and mental health, health behaviour and psychosocial variables were measured. Multinomial logistic regression analyses were used to estimate determinants of loneliness and logistic and linear regression analyses were applied to estimate the associations of loneliness with physical, mental and health risk behaviour variables.

**Results:**

The self-reported prevalence of loneliness (occasionally or all of the time or 3–7 days per week) was 10.6% (11.0% for females and 10.1% for males), and 8.0% reported sometimes (1–2 days/week) to be lonely. Loneliness was distributed in a slight U-shaped form, with adolescents and the oldest old having the highest prevalence of loneliness. In adjusted multinomial logistic regression analysis, lower education, lower economic status, adverse childhood experiences, having one or more chronic conditions, functional disability and low neighbourhood trust were associated with loneliness. Loneliness was significantly associated with most health variables, including self-reported unhealthy health status (AOR 1.70, CI 1.57, 1.84), cognitive functioning (Beta: − 0.72, CI − 0.90 to − 0.54), having one or more chronic medical conditions (AOR 1.25, CI 1.16, 1.35), having had a stroke (AOR 1.58, CI 1.08, 2.29), depression symptoms (Beta: 5.19, CI 4.98–5.39), sleep disturbance (Beta: 0.34, CI 0.31–0.37), sleep related impairment (Beta: 0.69, CI 0.64–0.73), low life satisfaction (AOR 1.78, CI 1.64, 1.93), out-patient health care utilization in the past 4 weeks (AOR 1.11, CI 1.01, 1.21), current tobacco use (AOR 1.42, CI 1.28, 1.58), and one or more days in the past week soft drink consumption (AOR 1.20, CI 1.10, 1.31).

**Conclusion:**

Loneliness was found to be prevalent across the life span and was associated with a number of poorer health variables. Several factors associated with loneliness were identified, which warrant further research in Indonesia.

## Introduction

According to Hawkley and Cacioppo ([1], p. 218), loneliness is a “distressing feeling that accompanies the perception that one’s social needs are not being met by the quantity or especially the quality of one’s social relationships.” A loneliness model by Hawkley and Cacioppo ([1], p. 222) “posits that perceived social isolation is tantamount to feeling unsafe, and this sets off implicit hypervigilance for (additional) social threat in the environment. Unconscious surveillance for social threat produces cognitive biases: relative to nonlonely people, lonely individuals see the social world as a more threatening place, expect more negative social interactions, and remember more negative social information. Negative social expectations tend to elicit behaviors from others that confirm the lonely persons’ expectations, thereby setting in motion a self-fulfilling prophecy in which lonely people actively distance themselves from would-be social partners even as they believe that the cause of the social distance is attributable to others and is beyond their own control. This self-reinforcing loneliness loop is accompanied by feelings of hostility, stress, pessimism, anxiety, and low self-esteem and represents a dispositional tendency that activates neurobiological and behavioral mechanisms that contribute to adverse health outcomes.”

Loneliness occurs across the life span, yet most studies investigated loneliness during older age and adolescents in high-income countries, and only few studies studied loneliness across the life span, including Asian countries [2–9]. An increasing number of studies seem to show negative effects of loneliness on physical and mental health as well as health behaviour. Studies showed that loneliness was associated with poor self-reported health status [5, 6, 10, 11]. Other studies show a negative effect of loneliness on physical health, such as self-reported chronic diseases [5], hypertension [12, 13], increased vulnerability to stroke, cardiovascular diseases [14–16], diabetes [5]. Further, a variety of studies found an association between loneliness and poor mental health such as poor sleep quality and greater sleep disturbance [10, 17, 18], mental health problems, such as depression [5, 9, 19, 20], psychological distress [5, 6] and low life satisfaction [21]. Greater loneliness was found to be associated with lower cognitive functioning [22]. The risk of unhealthy behaviours was found to be higher among lonely than non-lonely individuals such as tobacco use [5, 10, 19, 23] physical inactivity [24], including having obesity [25, 26], inadequate fruit and vegetable consumption [5] and consumption of sugary beverages [27]. Several studies also found that loneliness had been associated with health-care utilisation [5, 19, 28], while another study among older adults in Singapore found a negative association [29].

The prevalence of experiencing loneliness varied by country. In a national survey among the general adult population in Germany, the prevalence of some loneliness was 10.5% (4.9% slight, 3.9% moderate and 1.7% severe) [19]. In the general adult population in Switzerland, 31.7% felt sometimes and 4.3% quite often or very often lonely [5]. In countries of the former Soviet Union, the prevalence of (often) loneliness among the general adult population ranged from 4.4% in Azerbaijan to 17.9% in Moldova [6]. In a national sample of adolescents in Indonesia, 9.6% of students reported mostly or always feeling lonely in the past year [7]. In Malaysia nearly one-third of older adults reported a lot of loneliness [13].

Some sociodemographic characteristics seem to increase the risk of having loneliness. Regarding gender, mixed results were found, with some studies finding a higher prevalence of loneliness among adolescent boys or adult men [30, 31] and other studies among adolescent girls or adult women [3, 19, 31, 32]. Regarding age, several studies found a non-linear U-shaped prevalence of loneliness, with more lonely younger and older or very old individuals than in middle-aged adults [3–5, 33], while other studies found different variations of loneliness prevalence across the life span, including an increase or decline of loneliness with age [2, 6, 19, 32]. Several studies found an association between lower socioeconomic status [34], lower economic [10, 32] and lower educational status [32] and loneliness. Adverse childhood experiences [10, 35, 36] have also been found to associated with adult loneliness. On the other hand, social support [6, 10, 20, 31], being married [6, 19], social capital (high levels of trust) [37], and social engagement [9] seems protective against loneliness.

Indonesia has been undergoing rapid socioeconomic transition, a growing population and rapid urbanisation [38], social transition (e.g., greater proportion of singles or never married) [39], greater mobile phone, internet, and media exposure [40], and loneliness among the left-behind children of migrant workers in Indonesia [41]. Afandi [42] notes that “Indonesia is the country with the highest level of the social gap in Asia. It is predictable that one contributing factor is the gentrification that recently occurs rapidly in major cities in Indonesia… and social distance can be a predictor of various social chaos and conflict in the community.” For example, “My parents don’t have much time for me because they are busy with work. I feel lonely. I don’t have that closeness with my parents and friends. I felt like I don’t have (real) friends and sometimes think my friends don’t like me” [42]. “The issue of loneliness is especially significant in Indonesia” [42]. “In traditional society, it was unusual for people to be alone, and being by oneself is still considered both undesirable and also inappropriate” [42]. “However, rapid social change, including changes in employment, and the time pressures and travel distances have changed the pattern of many people’s daily life” [42]. “As a result, loneliness is an increasing problem for all age groups, and it is a source of stress that a majority of Indonesians are unequipped to deal with” [8, 42]. For example, low social skills were associated with increased loneliness in university students in Indonesia [43].

Loneliness has been recognized as an important public health issue [44–46], and as it is associated with stigma, services for lonely people are difficult to implement due to the difficulty to identify or reach them [47]. It is hoped that this population-based study in the general population in Indonesia may help to identify risk populations so as to provide informed prevention and intervention efforts for loneliness [48]. Considering the paucity of data on loneliness or its association with health in Southeast Asian countries, including Indonesia, the aim of this study is to estimate the prevalence of loneliness, its correlates and associations with health variables in a national survey in the general population in Indonesia.

## Methods

### Sample and procedure

Cross-sectional national data (representing 83% of the population) were analysed from the 2015 “Indonesia Family Life Survey (IFLS-5)”, details of the survey methodology have been described elsewhere [49]. The response rate was over 90% [49]. The IFLS-5 has been approved by ethics review boards of RAND and University of Gadjah Mada in Indonesia [49]. Written informed consent was obtained from all respondents prior to data collection.

### Measures

The *loneliness* question used for this analysis comes from the Center for Epidemiologic Studies Depression Scale (CES-D-10) [50]: “How often did you feel lonely in the past week?” Response options were 1 = rarely or none of the time (< 1 day), 2 = Some or a little of the time (1–2 days), 3 = Occasionally or a moderate amount of time (3–4 days), or 4 = All of the time (5–7 days) [50]. This single item measure has been used previously [51], and significantly correlates (r = 0.79, P < 0.001) with the UCLA Loneliness Scale [52]. The remaining 9 items of the CES-D-10 were used to assess depression symptoms, scored 0–3 for each item, total scores ranging from 0 to 27 [50]; Cronbach’s alpha was 0.66 in this study.

*Sociodemographic variables* included age, sex, education, residential status, and subjective socioeconomic background [49].

*Childhood adversity* questions included: (1) “Would you say that your health during your childhood was in general excellent, very good, good, fair, or poor?” (2) “Did you experience hunger in your childhood (from birth to 15 years)?” [49].

*Chronic medical conditions* were assessed with the question, “Has a doctor/paramedic/nurse/midwife ever told you that you had…?” (“Hypertension, Diabetes or high blood sugar, Heart attack, coronary heart disease, angina or other heart problems, Stroke, tuberculosis, asthma, other lung conditions, liver, cancer or malignant tumour, arthritis/rheumatism, and uric acid/gout.”) (Yes, No) [49]. All chronic medical conditions were summed up to indicate if an individual had no, one or two or more medical conditions.

*Functional disability* was measured with Instrumental Activities of Daily Living (= IADL) (6 items) [53]. (Cronbach’s alpha 0.91). A dichotomized functional disability total score was constructed and IADL disability classified as having difficulty in one or more IADL items.

*Social capital* was assessed with 4 items: “During the last 12 months did you participate or use?…” (1) Community meeting, (2) Voluntary labour, (3), Programme to improve the village/neighbourhood, and (4) Religious activities. Response options, were “yes” or “no” [49]. (Cronbach’s alpha 0.69). Participants that never scored with “yes” were classified as having low social capital.

*Neighbourhood trust* was assessed with two items, e.g., *“*In most parts of the community or village, is it safe for you to walk alone at night?” [49]. Response options ranged from 1 = very unsafe to 4 = very safe, which were summed giving scores from 2 to 8.

*Self*-*reported health status* was measured with the question, “In general, how is your health?” Response options were 1 = Very healthy, 2, Somewhat healthy, 3 = Somewhat unhealthy, and 4 = Unhealthy [49].

*Cognitive functioning* was assessed with questions from the Telephone Survey of Cognitive Status (TICS) [54]. Total scores ranged from 0 to 34.

#### Hypertension measurement and classification

Three consecutive measurements of systolic and diastolic blood pressure (BP) were averaged. “Hypertension was defined as SBP ≥ 140 mm Hg and/or DBP ≥ 90 mm Hg and/or current use of antihypertensive medication. Normotension was defined as BP values < 120/80 mm Hg in individuals who were not taking antihypertensive medication” [55].

*Sleep disturbance* was assessed with five items from the “Patient-Reported Outcomes Measurement Information System (PROMIS)” sleep disturbance measure [56]. Response options ranged from 1 = not at all to 5 = very much. (Cronbach’s alpha = 0.68).

*Sleep Related Impairment* was assessed with five items from the PROMIS sleep impairment measure [57]. Response options ranged from 1 = not at all to 5 = very much. (Cronbach’s alpha = 0.82). For both sleep measures the five items were summed giving scores from 5 to 25 and total item mean scores from 1 to 5.

*Life satisfaction* was assessed with the question, “Please, think about your life as a whole. How satisfied are you with it?” Response options ranged from 1 = completely satisfied to 5 = not at all satisfied [49]. Low life satisfaction was defined as not very or not at all satisfied.

*Health care utilization* was assessed with the question, “Whether visited any outpatient health care clinic in 1 month prior to survey or not?”(Yes, No) [49].

*Anthropometric measurements.* Body mass index (BMI) was calculated as measured weight in kg divided by measured height in metre squared [58].

*Tobacco use* was assessed with two questions: (1) “Have you ever chewed tobacco, smoked a pipe, smoked self-enrolled cigarettes, or smoked cigarettes/cigars?” (Yes, No), (2) “Do you still have the habit or have you totally quit?”(Still have, Quit) [49]. Responses were grouped into never or quitters and current tobacco users.

*Physical activity* was assessed with a modified version of the “International Physical Activity Questionnaire (IPAQ) short version, for the last 7 days (IPAQ-S7S)”. We used the instructions given in the IPAQ manual [59], and categorized physical activity according to the IPAQ procedures [60] as low, moderate and high (low = physical inactivity).

*Fruit and vegetable consumption* was assessed with questions on, “How many days in the past week did you eat, (1a) Green leafy vegetables? (1b) carrots? (2a) banana? (2b) papaya? (2c) mango?” [49]. Infrequent fruit consumption was defined as less than 3 days a week, and infrequent vegetable consumption as less than daily.

*Soft drink consumption* was assessed with the question, “How many days in the past week did you have a soft drink (Coca cola, sprite, etc.)?” [49] (Coded as any day of the week).

### Data analysis

Descriptive statistics were calculated to describe the sample. First unadjusted, followed by adjusted multinomial logistic regression was used determine the relative risk ratio (RRR) and 95% confidence intervals (CIs) between socio-demographic factors, childhood adversity, having chronic conditions, functional disability, social capital and loneliness status. The dependent variables were moderate loneliness (sometimes or 1–2 days/week) and severe loneliness (occasionally or all the time or 3–7 days/week) and the comparison group, individuals with rarely or no (< 1 day/week) loneliness. Unconditional logistic regression and linear regression analyses were utilized to estimate the associations of loneliness (occasionally or all the time or 3–7 days/week) with health status, physical and mental health and health risk behaviour variables in three models (model 1 unadjusted, model 2 age- and sex adjusted, and model 3 adjusted for age, sex, marital status, residence, economic status, education, social capital, and neighbourhood trust [5]. Potential multi-collinearity between variables was assessed with variance inflation factors, none of which exceeded critical value. *P* < 0.05 was considered significant. “Cross-section analysis weights were applied to correct both for sample attrition from 1993 to 2014, and then to correct for the fact that the IFLS1 sample design included over-sampling in urban areas and off Java. The cross-section weights are matched to the 2014 Indonesian population, again in the 13 IFLS provinces, in order to make the attrition-adjusted IFLS sample representative of the 2014 Indonesian population in those provinces” [49]. Both the 95% confidence intervals and P-values were adjusted considering the survey design of the study. All analyses were done with STATA software version 13.0 (Stata Corporation, College Station, TX, USA).

## Results

### Sample characteristics and prevalence of loneliness

The total sample included 31,447 individuals 15 years and older, males = 49.2%; median age 35.0 years, IQR = 22.0, age range of 15–109 years, from Indonesia. The self-reported prevalence of loneliness (occasionally or all of the time or 3–7 days per week) was 10.6% (11.0% for females and 10.1% for males), and 8.0% reported sometimes (1–2 days/week) to be lonely (see Table 1).

**Table 1:** Sample characteristics

Variable	Variable specification	Total
Total, n		31,447
Age, median (IQR)		35.0 (22.0)
Sex	Males	49.2%
	Females	50.8%
Age	15–29	21.9%
	30–59	58.8%
	60 or more	19.3%
Education	High school or higher	58.1%
	None or elementary	41.9%
Subjective economic background	Poor	24.8%
	Medium	46.7%
	Rich	28.5%
Marital status	Unmarried	16.4%
	Married, cohabitating	72.9%
	Separated/divorced/widowed	10.6%
Residence	Urban	53.4%
Childhood hunger	Yes	8.4%
Childhood health status	Poor/fair	37.2%
	Good	40.2%
	Very good	22.6%
Chronic conditions	One or more	35.1%
Instrumental activities of daily living	Yes	23.7%
Social capital	Low	41.5%
Neighbourhood trust	Scale	M = 6.0 (SD = 0.8)
Self-rated status	Unhealthy	21.9%
Cognitive functioning	Scale	M = 18.8 (SD = 4.6)
Hypertension^a^	Yes	31.9%
Stroke	Yes	1.0%
Heart problems	Yes	1.9%
Diabetes	Yes	2.7%
Depression symptoms	Scale	M = 5.9 (SD = 4.4)
Sleep disturbance	Scale	M = 2.2 (SD = 0.7)
Sleep related impairment	Scale	M = 1.9 (SD = 0.9)
Life satisfaction	Low	14.6%
Out-patient visit in the past 4 weeks	Yes	18.9%
Body mass index^a^	Scale	M = 23.3 (SD = 4.6)
Tobacco use	Current	32.9%
Physical activity	Inactive	47.7%
Fruit and vegetable consumption	Infrequent	38.2%
Soft drink consumption	One day or more in a week	17.9%
Loneliness	Rarely or none of the time (< 1 day)	81.0%
	Some or a little of the time (1–2 days)	8.0%
	Occasionally/moderate amount of time (3–4 days)	7.3%
	All of the time (5–7 days)	3.3%

IQR, interquartile range; M, mean; SD, standard deviation

^a^In the sample 18 years and above

**Fig. 1:**
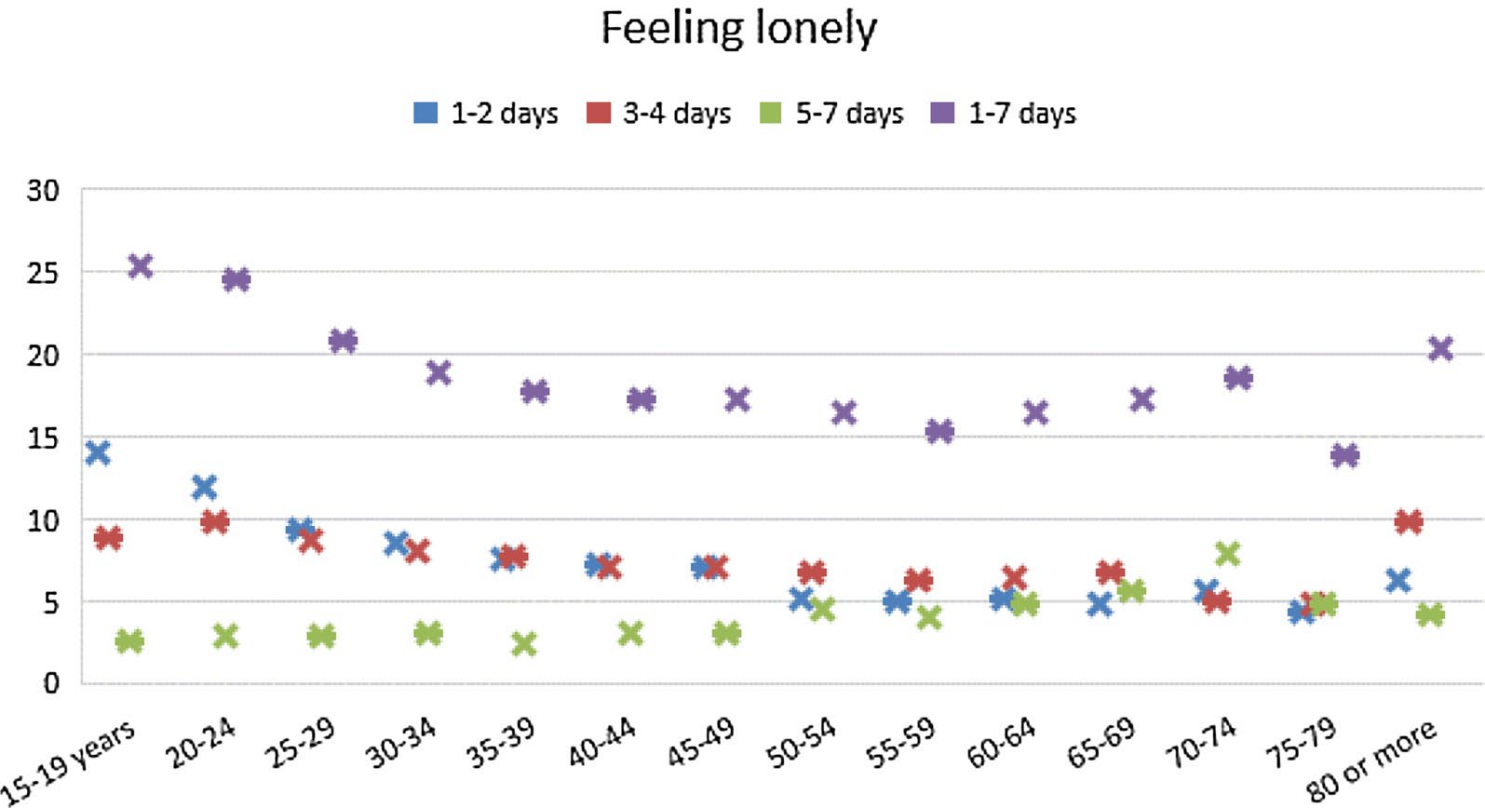
Prevalence of loneliness by age group

The prevalence of loneliness was highest in the age groups 15–24 years, followed by the oldest old (80 years or more) and the 70–74 years age group, while the lowest feelings of loneliness were reported among the 75–70 year-olds and the 55–59 year-olds (see Fig. 1).

#### Associations with loneliness

In adjusted multinomial logistic regression analysis, compared to 15–29 year-olds moderate loneliness was lower at middle and older age, while severe loneliness was lower at middle age but not at older age. Higher educational level and richer subjective economic background were negatively associated with both moderate and severe loneliness. Being married or cohabiting was negatively associated with both moderate and severe loneliness. Having experienced childhood hunger was associated with both moderate and severe loneliness. Better childhood health status decreased the odds of severe loneliness. Compared to individuals with no chronic medical condition, those with one or two or more chronic conditions were more likely to experience both moderate and severe loneliness. Having functional disabilities (IADL) were associated with a higher risk of moderate and severe loneliness. Social capital in terms of neighbourhood trust was protective from both moderate and severe loneliness (see Table 2).

**Table 2:** Predictors of loneliness

Variable	Moderate loneliness		Severe loneliness	
	CrRRR (95% CI)	ARRR (95% CI)	CrRRR (95% CI)	ARRR (95% CI)
Gender				
Female	1 (reference)	1 (reference)	1 (reference)	1 (reference)
Male	1.13 (1.05, 1.20)***	1.06 (0.96, 1.16)	0.92 (0.86, 0.99)*	0.93 (0.85, 1.02)
Age				
15–29	1 (reference)	1 (reference)	1 (reference)	1 (reference)
30–59	0.54 (0.50, 0.58)***	0.59 (0.52, 0.66)***	0.84 (0.78, 0.90)***	0.74 (0.66, 0.83)***
60 or more	0.41 (0.35, 0.47)***	0.37 (0.30, 0.46)***	1.01 (0.91, 1.13)	0.64 (0.53, 1.13)
Education				
None or elementary	1 (reference)	1 (reference)	1 (reference)	1 (reference)
High school or higher	0.80 (0.74, 0.87)***	0.85 (0.76, 0.94)**	0.60 (0.56, 0.64)***	0.60 (0.54, 0.66)***
Subjective economic background				
Poor	1 (reference)	1 (reference)	1 (reference)	1 (reference)
Medium	0.84 (0.77, 0.92)***	0.82 (0.73, 0.92)***	0.59 (0.55, 0.64)***	0.66 (0.60, 0.73)***
Rich	0.70 (0.64, 0.78)***	0.69 (0.60, 0.79)***	0.56 (0.51, 0.61)***	0.65 (0.57, 0.72)***
Marital status				
Unmarried	1 (reference)	1 (reference)	1 (reference)	1 (reference)
Married, cohabitating	0.50 (0.46, 0.55)***	0.72 (0.63, 0.82)***	0.78 (0.72, 0.85)***	0.77 (0.67, 0.87)***
Separated/divorced/widowed	0.57 (0.49, 0.66)***	0.92 (0.73, 1.14)	1.23 (1.09, 1.39)***	1.05 (0.86, 1.28)
Residence				
Rural	1 (reference	1 (reference)	1 (reference)	1 (reference)
Urban	0.99 (0.92, 1.07)	1.02 (0.92, 1.11)	0.87 (0.81, 0.93)***	0.97 (0.89, 1.05)
Childhood hunger				
No	1 (reference)	1 (reference)	1 (reference)	1 (reference)
Yes	1.29 (1.14, 1.46)***	1.36 (1.15, 1.61)***	1.82 (1.65, 2.01)***	1.52 (1.33, 1.73)***
Childhood health status				
Poor/fair	1 (reference)	1 (reference)	1 (reference)	1 (reference)
Good	0.86 (0.79, 0.93)***	0.89 (0.80, 0.99)*	0.76 (0.70, 0.82)***	0.81 (0.74, 0.89)***
Very good	0.89 (0.81, 0.98)*	0.90 (0.79, 1.01)	0.89 (0.82, 0.97)**	0.96 (0.86, 1.06)
Chronic conditions				
None	1 (reference)	1 (reference)	1 (reference)	1 (reference)
One	1.12 (1.03, 1.22)*	1.12 (1.01, 1.25)*	1.18 (1.09, 1.28)***	1.20 (1.09, 1.32)***
Two or more	1.22 (1.08, 1.39)**	1.22 (1.04, 1.43)*	1.28 (1.15, 1.41)***	1.32 (1.15, 1.51)***
IADL				
No	1 (reference)	1 (reference)	1 (reference)	1 (reference)
Yes	1.65 (1.52, 1.79)***	1.42 (1.28, 1.57)***	1.56 (1.45, 1.67)***	1.41 (1.28, 1.54)***
Social capital				
Low	1.18 (1.07, 1.29)***	1.03 (0.93, 1.13)	1.08 (0.99, 1.17	1.03 (0.94, 1.13)
Neighbourhood trust				
Scale	0.91 (0.86, 0.96)***	0.92 (0.87, 097)**	0.90 (0.85, 0.94)***	0.90 (0.86, 0.95)***

IADL, instrumental activities of daily living *** P < 0.001; ** P < 0.01; * P < 0.05; CrRRR, crude relative risk ratio; ARRR, adjusted relative risk ratio

#### Associations between loneliness and health variables

In adjusted logistic or linear regression models, loneliness was associated with self-reported unhealthy health status (AOR 1.70, CI 1.57, 1.84), lower cognitive functioning (Beta: − 0.72, CI − 0.90 to − 0.54), having one or more chronic medical condition (AOR 1.25, CI 1.16, 1.35), having had a stroke (AOR 1.58, CI 1.08, 2.29), depression symptoms (Beta: 5.19, CI 4.98, 5.39), sleep disturbance (Beta: 0.34, CI 0.31, 0.37), sleep related impairment (Beta: 0.69, CI 0.64, 0.73), low life satisfaction (AOR 1.78, CI 1.64, 1.93), out-patient health care utilization in the past 4 weeks (AOR 1.11, CI 1.01, 1.21), current tobacco use (AOR 1.42, CI 1.28, 1.58), and once or more days in the past week soft drink consumption (AOR 1.20, CI 1.10, 1.31). Furthermore, loneliness was statistically negatively associated with BMI (Beta: 0.35, CI − 0.52, − 0.18) and physical inactivity (AOR 0.92, CI 0.85, 0.98) (see Table 3).

**Table 3:** Multivariable logistic regression analyses of the association between loneliness and health variables

Variable (outcome)	Variable response	Model 1: unadjusted odds ratio or beta (95% CI)	Model 2: adjusted odds ratio or beta (95% CI)^b^	Model 3: adjusted odds ratio or beta (95% CI)^c^
Health status				
Unhealthy self-rated status	No	1 (reference)	1 (reference)	1 (reference)
	Yes	1.74 (1.62, 1.87)***	1.81 (1.65, 1.99)***	1.70 (1.57, 1.84)***
Cognitive functioning	Scale	− 1.17 (− 1.37 to − 0.96)***	− 1.18 (− 1.37 to − 0.99)***	− 0.72 (− 0.90 to − 0.54)***
Chronic medical conditions	None	1 (reference)	1 (reference)	1 (reference)
	One or more	1.20 (1.10, 1.31)***	1.20 (1.10, 1.31)***	1.25 (1.16, 1.35)***
Hypertension^a^	No	1 (reference)	1 (reference)	1 (reference)
	Yes	1.10 (1.01, 1.21)*	1.08 (0.98, 1.20)	1.07 (0.99, 1.17)
Stroke	No	1 (reference)	1 (reference)	1 (reference)
	Yes	1.66 (1.05, 2.63)*	1.58 (0.99, 2.50)	1.58 (1.08, 2.29)*
Heart problems	No	1 (reference)	1 (reference)	1 (reference)
	Yes	1.33 (1.00, 1.76)*	1.28 (0.97, 1.70)	1.15 (0.89, 1.50)
Diabetes	No	1 (reference)	1 (reference)	1 (reference)
	Yes	0.89 (0.67, 1.18)	0.84 (0.63, 1.13)	1.02 (0.81, 1.29)
Depression symptoms	Scale	5.34 (5.13 to 5.54)***	5.33 (5.13 to 5.53)***	5.19 (4.98 to 5.39)***
Sleep disturbance	Scale	0.35 (0.32 to 0.38)***	0.35 (0.32 to 0.38)***	0.34 (0.31 to 0.37)***
Sleep related impairment	Scale	0.71 (0.66 to 0.75)***	0.70 (0.66 to 0.75)***	0.69 (0.64 to 0.73)***
Low life satisfaction	No	1 (reference)	1 (reference)	1 (reference)
	Yes	2.03 (1.87, 2.19)***	2.04 (1.89, 2.21)***	1.78 (1.64, 1.93)***
Out-patient visit in the past 4 weeks	No	1 (reference)	1 (reference)	1 (reference)
	Yes	1.09 (1.00, 1.18)*	1.09 (0.98, 1.21)	1.11 (1.01, 1.21)*
Body mass index^a^	Scale	− 0.59 (− 0.77 to − 0.41)***	− 0.56 (− 0.73 to − 0.39)***	− 0.35 (− 0.52 to − 0.18)***
Tobacco use	Never/former	1 (reference)	1 (reference)	1 (reference)
	Current	1.16 (1.06, 1.77)***	1.52 (1.38, 1.68)***	1.42 (1.28, 1.58)***
Physical activity	Moderate/high	1 (reference)	1 (reference)	1 (reference)
	Inactive	90.5 (0.83, 0.98)*	0.90 (0.83, 0.98)*	0.92 (0.85, 0.98)*
Fruit and vegetable consumption	Frequent	1 (reference)	1 (reference)	1 (reference)
	Infrequent	1.10 (1.01, 1.24)*	1.09 (1.00, 1.18)*	1.04 (0.96, 1.12)
Soft drink consumption	No days/week	Reference	Reference	Reference
	1–7 days/week	1.12 (1.03, 1.21)**	1.15 (1.05, 1.25)**	1.20 (1.10, 1.31)***

^a^In the sample 18 years and above, ^b^ adjusted for age and sex, ^c^ adjusted for age, sex, marital status, residence, economic status, education, social capital, and neighbourhood trust

## Discussion

This is the first study investigating loneliness correlates and associations with health variables in a national sample of the general population in Southeast Asia, in Indonesia. The study found a considerable prevalence of loneliness in this general population in Indonesia, which was higher than in a previous study in the general population in Germany [19], lower than among older adults in Malaysia [13] and the general population in Switzerland [5], and similar to a study in the general population across nine countries of the former Soviet Union [6] and similar to a national sample of school-going adolescents in Indonesia [7]. Previous studies in Indonesia [38–43] have identified the importance of loneliness in different age groups of the population, and various factors, such as rapid socioeconomic change, urbanization, migration, gentrification, and modern media penetration, may be attributed to the development of loneliness or social isolation in Indonesia.

There was no significant difference in the prevalence of loneliness among females and males. Other studies also found mixed results regarding sex differences [3, 19, 30–32]. Regarding the prevalence distribution of loneliness across the life span, this study found that loneliness was distributed in a slight U-shaped form, with adolescents and the oldest old having the highest prevalence of loneliness. Several other studies also found a non-linear U-shaped distribution [3–5, 33], emphasising the importance of loneliness among the young and older aged populations.

In agreement with previous studies [3, 10, 32, 35, 36], this study found that lower economic status, lower educational level, rural residence and adverse childhood experiences were associated with adult loneliness. Persons from lower socioeconomic backgrounds may have less resources and opportunities that could prevent them becoming lonely [32]. Future research may investigate possible pathways that may be responsible for the association of adverse childhood experiences and adult loneliness [36]. Similarly, this study found, as also previously found [6, 10, 19, 20, 31, 37], that better social support, being married and higher social capital in terms of trust were protective against loneliness. Having one or more chronic condition and functional disability were also in this study found to be associated with loneliness. This may be explained by the limiting effect of having chronic conditions and/or having functional disability on the participation and performance of specific activities [61]. These findings suggests that loneliness interventions should target individuals with these socioeconomic characteristics, those with functional disability and those with low social capital (trust).

This study confirmed findings from previous studies [5, 6, 9–11, 14, 17–20] of associations between loneliness and a number of physical and mental health variables, including self-reported unhealthy health status, low cognitive functioning, having one or more chronic medical condition, having had a stroke, depression symptoms, sleep disturbance, sleep related impairment, and low life satisfaction. The high association between loneliness and depression symptoms in this study may be explained by the high accompaniment of loneliness in depression, being part of depression symptomatology and being both a risk factor and consequence of depression [5]. Unlike some other studies [5, 12, 13, 15, 16], this study did not find an association between loneliness and hypertension, heart problems and diabetes. As found in several previous studies [5, 19, 28], this study found that loneliness had been associated with health-care utilisation. It is possible that lonely individuals have poorer health and therefore need to see the health care provider more often than non-lonely individuals [5]. Moreover, seeing and talking to a health care provider may take care of overcoming social isolation or loneliness [5, 62]. In addition, this study found in agreement with previous studies [5, 10, 19, 23, 27], an association between loneliness and lifestyle factors, including tobacco use, soft drink consumption and marginally inadequate fruit and vegetable consumption. We observed the association between loneliness and tobacco use, independently of age, so that tobacco use may be used as a method to connect with others in order to reduce loneliness across the life span. The association between loneliness and soft drinks consumption seems to confirm social baseline theory that social isolation influences higher levels of sugar consumption [27].

Contrary to some previous studies [24–26] that found an association between loneliness and higher BMI and physical inactivity, this study found a negative relationship. In another study in Indonesia, a negative association between depression and having overweight or obesity was found [63]. It is possible that having higher BMI or obesity in Indonesia is associated with improved socioeconomic status and ideal body image symbolising nurturance and affluence [64] associated with reduced loneliness. It is possible that physical inactivity is seen similarly to having higher BMI or obesity in this transitional Indonesian society as something to be aspired to, such as having a higher paid office job than a lower paid manual labour job associated with more physical activity.

There might be several possible pathways of linking loneliness with poor health [46]. For example, poor self-rated health status can co-occur with sleep disturbance and sleep related impairment and may reinforce each other over time. Loneliness may generate anxiety-related thoughts that hamper relaxation resulting in sleep disturbance and impairment [46, 64]. Moreover, the study found an association between different stressors (childhood adversity, poor socioeconomic status) and loneliness. Stress could be linking loneliness with poor health [65]. Lonely persons may have a heightened perception of stress, anxiety, depression and mistrust, which activate “neurobiological and behavioral mechanisms that contribute to adverse health outcomes [1].”

### Study limitations

The study was cross-sectional in design, so causal conclusions cannot be drawn. As the questionnaire part of the study relied on self-report, so response bias is a possibility. The questionnaire used in this study assessed loneliness with a single item. However, a high correlation between single-item and multi-item loneliness indices has been found [63]. Further, we interpreted the more frequent loneliness experience as the more serious as the less frequent experience of loneliness [6]. Future research should also measure the intensity of the loneliness experience. Certain variables that may contribute to the understanding of loneliness, such as household size (living alone) and personality related factors, were not assessed in this study, and should be included in future research.

## Conclusions

Loneliness was found to be prevalent across the life span and was associated with a number of poorer physical and mental health variables and health risk behaviours. Several factors found in this study to be associated with loneliness were identified, such as low socioeconomic status, rural residence, adverse childhood experiences, having chronic conditions, functional disability and lack of neighbourhood trust, warrant further research in Indonesia.

## Abbreviations

BMI: body mass index
CES-D: Centres for Epidemiologic Studies Depression Scale
EA: enumeration area
IFLS: Indonesian Family Life Survey
IPAQ: International Physical Activity Questionnaire
